# Positive Effect of a Successfully Implemented Model of Care on Unplanned Transfers to Hospital

**DOI:** 10.1093/geroni/igab046.2124

**Published:** 2021-12-17

**Authors:** Franziska Zuniga, Raphaëlle Guerbaai, Michael Simon

**Affiliations:** 1 Basel University, Ostermundingen, Basel-Stadt, Switzerland; 2 Institute of Nursing Science, Department of Public Health, University of Basel, Institute of Nursing Science, Department of Public Health, University of Basel, Basel-Stadt, Switzerland; 3 University of Basel, Basel, Basel-Stadt, Switzerland

## Abstract

Models of care have shown effectiveness in reducing unplanned transfers in nursing homes (NHs) from 11.7% to 6.1%. These include coordination of care and access to skilled medical providers such as geriatricians, specialist nurses or registered nurses with additional training. A hybrid type-2 effectiveness-implementation project (INTERCARE) was developed to improve intervention uptake and to understand the mechanisms behind results. INTERCARE consisted of six core elements and was rolled-out to 11 Swiss NHs with a stepped-wedge design allowing all NHs to receive the intervention. 942 residents were recruited (June 2018 -January 2020). INTERCARE showed a significant reduction of unplanned transfers during the intervention period compared with baseline. The successful implementation of INTERCARE relied on the use of implementation science, building on stakeholder input and careful theory-driven contextual adaptations. INTERCARE’s success was driven by registered nurses with expanded roles, on-site coaching, and the use of tools for clinical decision making.

